# *Fagopyrum cymosum* Extract Improves Mitochondrial Function and Reduces Colonic Inflammation in Ulcerative Colitis by Targeting the PI3K/Akt/FOXO3A Pathway

**DOI:** 10.3390/biomedicines14102403

**Published:** 2026-10-21

**Authors:** Shanshan Xu, Congtian Fan, Haidong Chen, Dan Chen, Jie Lu, Jianyong Xu, Hua Huang, Yu Jiang

**Affiliations:** 1 Nantong Hospital Affiliated to Nanjing University of Chinese Medicine, Nantong 226001, China; symphoni33@163.com (S.X.);; 2 Changshu Hospital Affiliated to Nanjing University of Chinese Medicine, Changshu 215500, China

**Keywords:** ulcerative colitis, *Fagopyrum cymosum*, PI3K/Akt/FOXO3A signaling pathway, mitochondrial function, oxidative stress, intestinal inflammation

## Abstract

**Background/Objectives:** Ulcerative colitis (UC) is a chronic inflammatory bowel disease with limited therapeutic options. Although *Fagopyrum cymosum* exhibits anti-inflammatory and antioxidant activities, its efficacy in UC and the underlying mechanisms are not well defined. Here, we evaluate whether *Fagopyrum cymosum* extract (RFC) alleviates DSS-induced colitis in mice and investigate the involvement of the PI3K/Akt/FOXO3A pathway. **Methods:** C57BL/6 mice received 2% DSS for 14 days, followed by 7-day intragastric RFC (low: 234 mg/kg; medium: 468 mg/kg; high: 936 mg/kg) with 5-ASA (200 mg/kg) as a control. Disease activity index (DAI), colon length, histopathology, bacterial translocation, and organ indices were assessed. Colonic mitochondrial membrane potential (JC-1), ATP, oxidative markers (SOD, GSH-px, MDA), and cytokines (IL-6, IL-1β, TNF-α) were measured. Network pharmacology, qPCR, and Western blotting were performed, alongside a PI3K agonist (740Y-P) rescue experiment. **Results:** RFC dose-dependently improved weight loss, DAI, colon shortening, mucosal damage, bacterial loads, and immune organ indices. It restored mitochondrial function, reduced MDA, elevated SOD/GSH-px, and suppressed inflammatory cytokines. Mechanistically, RFC inhibited PI3K/Akt phosphorylation and upregulated FOXO3A, effects reversed by 740Y-P, which also abolished RFC’s protective, antioxidant, and anti-inflammatory actions. **Conclusions:** RFC ameliorates DSS-induced colitis by suppressing PI3K/Akt signaling and upregulated FOXO3A expression, thereby preserving mitochondrial function, reducing oxidative stress, and alleviating colonic inflammation. The PI3K/Akt/FOXO3A axis emerges as a promising therapeutic node, and RFC merits further evaluation for UC treatment.

## 1. Introduction

Ulcerative colitis (UC) is a chronic relapsing inflammatory bowel disease that affects millions of people worldwide. Patients present with abdominal pain, chronic diarrhoea, rectal bleeding and weight loss [1,2]. Current treatments—including 5-aminosalicylic acid (5-ASA), glucocorticoids, immunosuppressants and biologics—can control acute flares, but long-term use frequently causes steroid dependence, increased infection risk and other adverse reactions [3]. Hence, safe natural medicines suitable for prolonged use are needed.

*Fagopyrum cymosum* (gold buckwheat), a Polygonaceae herb, is widely used in traditional Chinese medicine. Its main constituents are flavonoids, polyphenols and terpenoids, which confer anti-inflammatory, antioxidant and antimicrobial activities [4,5]. Although the herb has been traditionally used for pulmonary infections, a study using TNBS-induced colitis demonstrated that *F. cymosum* extract ameliorated body weight loss and colonic shortening, suppressed TNF-α and IL-6 production, and inhibited NF-κB p65 nuclear translocation and IκB phosphorylation [6]. These findings support the therapeutic potential of *F. cymosum* in UC. However, the mechanism by which RFC protects against UC remains incompletely understood, and whether the PI3K/Akt/FOXO3A pathway, a key regulator of mitochondrial function, oxidative stress and intestinal inflammation is involved has not been tested. Two lines of evidence suggest that RFC constituents may act through the PI3K/Akt pathway. First, quercetin, the aglycone of rutin which is abundant in *F. cymosum*, has been identified through network pharmacology as a key anti-UC component, with PI3K-AKT signaling as the most enriched pathway, and has been shown to inhibit PI3K/AKT phosphorylation in vitro [7]. Second, procyanidin B2, a major constituent detected in our RFC batch, protects against TNBS-induced colitis by suppressing PI3K/Akt phosphorylation, reducing pro-inflammatory cytokines, and restoring tight junction protein expression [8]. In addition, epicatechin, another major constituent of RFC, protects against DSS-induced colitis by enhancing antioxidant enzyme activity. The PI3K/Akt/FOXO3A axis is a key regulator of mitochondrial function, oxidative stress and intestinal inflammation in UC [9,10,11]. To identify whether RFC acts through this pathway, we performed network pharmacology analysis using TCMSP and SEA databases to predict RFC targets, intersected them with UC-associated genes from GeneCards and OMIM, and identified PI3K/Akt/FOXO3A as a top enriched pathway (Section 2.13). We then tested this prediction experimentally in DSS-induced colitis. However, whether RFC alleviates UC through this axis to improve mitochondrial function, reduce oxidative stress and suppress intestinal inflammation remains unknown [12,13].

Here, we combined a DSS-induced mouse colitis model with UHPLC-Q-TOF-MS/MS profiling, network pharmacology and PI3K-agonist rescue experiments to determine whether RFC protects against UC via the PI3K/Akt/FOXO3A pathway. This work provides experimental evidence supporting RFC as a potential UC therapeutic and identifies this signaling cascade as a functionally relevant target for further investigation.

## 2. Materials and Methods

### 2.1. Animals

SPF-grade male C57BL/6 mice (6–8 weeks old, body weight 20 ± 2 g) were obtained from Jiangsu Jicui Yaokang Biotechnology Co., Ltd. (Nanjing, Jiangsu, China). Mice were housed under SPF barrier conditions (22–24 °C, 50–60% humidity, 12-h light–dark cycle) with free access to sterilized chow and sterile water, following GB 14925-2010 standards [14]. All animal experiments were approved by the Animal Ethics Committee of Nantong University and conducted in compliance with the 3R principles.

### 2.2. Preparation and Chemical Profiling of Fagopyrum cymosum Extract (RFC)

Dried *Fagopyrum cymosum* material was ground, passed through a 10-mesh sieve, and extracted with 15 volumes of 65% ethanol (Sinopharm Chemical Reagent Co., Ltd., Shanghai, China) at 70 °C for 30 min under reflux. The extraction was repeated three times. The combined filtrates were pooled, concentrated under reduced pressure to remove ethanol, and dried to yield the RFC powder, following a previously described method [15].

The chemical constituents of the RFC batch used in the animal experiments were analyzed by UHPLC-Q-TOF-MS/MS (Bruker Daltonics, Bremen, Germany) with electrospray ionization in both positive and negative modes. Chromatographic separation and MS acquisition parameters are detailed in Supplementary Materials (Figure S1 and Table S1). The major tentatively annotated constituents were flavonoids (including epicatechin, procyanidin B2, and kaempferol glycosides), phenolic acids (such as gallic acid derivatives and syringic acid), and organic acids. Among the five candidate compounds retrieved from TCMSP network-pharmacology screening, only (-)-catechin gallate and gallate-containing proanthocyanidins were detected under the present analytical conditions; the remaining predicted components, including β-sitosterol and digallate, were not observed, likely owing to their relatively low abundance or suboptimal ionization efficiency. A complete list of tentative annotations is provided in Supplementary Table S1A,B.

### 2.3. DSS-Induced Colitis Model and Animal Grouping

Colitis was induced by administering 2% (*w*/*v*) DSS (MP216011, Thermo Fisher Scientific, Waltham, MA, USA) in sterile drinking water for 14 consecutive days, with fresh DSS solution replaced daily [16]. Control mice received sterile water ad libitum.

The study comprised two experimental sets. The first set included: control, DSS model, DSS + RFC-L (234 mg/kg), DSS + RFC-M (468 mg/kg), and DSS + RFC-H (936 mg/kg). The second set (pathway-rescue experiment) included: DSS model, DSS + RFC-H, DSS + RFC-H + 740Y-P (10 mg/kg, PI3K agonist), and DSS + 5-ASA (200 mg/kg, positive control). Doses were selected based on previously reported effective doses of *F. cymosum* extracts in rodent colitis models [6]. The LD50 of *F. cymosum* extract was greater than 5.0 g/kg in mice [6,17], and the maximum tolerated dose was 8.0 g/kg [18]. The selected doses (234, 468, and 936 mg/kg) fall within this safe range. All treatments were administered intragastrically once daily for 7 consecutive days after the 14-day DSS challenge.

### 2.4. Disease-Activity Index (DAI) Assessment

Body-weight changes, stool consistency and fecal occult blood were monitored daily. Fecal occult blood was detected using commercial kits (Nanjing Jiancheng Bioengineering Institute, Nanjing, China). DAI scores were calculated referring to published scoring criteria [19] (Table 1).

### 2.5. Colon-Length Measurement and Organ-Index Calculation

Mice were anesthetized with isoflurane (RWD Life Science, Shenzhen, China) and sacrificed by cervical dislocation. The full-length colon from the caecum terminus to the anus was isolated; fat and connective tissues were removed, and the colon length was measured. Mice were anesthetized with isoflurane and sacrificed by cervical dislocation. Body weight was recorded immediately before sacrifice. The full-length colon from the caecum terminus to the anus was isolated; fat and connective tissues were removed, and the colon length was measured. The spleen and thymus were excised, freed of adherent fat and connective tissue, rinsed in ice-cold PBS, blotted dry on filter paper, and weighed. Organ indices were calculated as wet organ weight (mg) divided by body weight before sacrifice (g). Spleen and thymus were selected as indicators of systemic immune activation, as splenomegaly and thymic atrophy are well-documented features of DSS-induced colitis in mice [16].

### 2.6. Bacterial-Load Determination in Mesenteric Lymph Nodes

Approximately 0.1 g of MLN tissue was homogenized in 0.9 mL ice-cold sterile saline. One hundred microliters of the homogenate was spread onto LB agar plates (BD Biosciences, Franklin Lakes, NJ, USA) and incubated at 37 °C for 24 h. Colony-forming units per gram of tissue (CFU/g) = colony count × dilution factor/tissue weight.

### 2.7. Mitochondrial-Membrane-Potential Detection by JC-1

Colonic tissues were washed with ice-cold PBS, cut into small pieces, and digested in RPMI-1640 (Gibco, Thermo Fisher Scientific, Waltham, MA, USA) medium containing 0.1% collagenase IV (Sigma-Aldrich, St. Louis, MO, USA) and 25 μg/mL DNase I (Roche, Basel, Switzerland) at 37 °C for 30 min with gentle agitation. The digested tissue was passed through a 70-μm cell strainer (BD Falcon, Franklin Lakes, NJ, USA), and the filtrate was centrifuged at 300× *g* for 5 min. The cell pellet was resuspended in PBS, and cell viability was assessed by trypan blue exclusion [20]. The JC-1 working solution was added and incubated at 37 °C for 20 min in darkness. After washing, red (JC-1 aggregate, high MMP) and green (JC-1 monomer, low MMP) fluorescence images were captured under an inverted fluorescence microscope (Olympus, Tokyo, Japan). JC-1 was excited at 488 nm, and emission was collected at 529 nm for green monomers and 590 nm for red J-aggregates. Image J software (version 1.53, NIH, Bethesda, MD, USA) was used to quantify the fluorescence intensity ratio; five random fields per sample were analysed [21].

### 2.8. Measurement of ATP, SOD, GSH-px and MDA

Colonic mucosal homogenates were prepared. The ATP content was assayed using commercial biochemical kits (Nanjing Jiancheng Bioengineering Institute, Nanjing, China). SOD and GSH-px activities and MDA levels were measured following the kit instructions. Read-outs were acquired via a multi-mode microplate reader (Molecular Devices, San Jose, CA, USA).

### 2.9. ELISA for Pro-Inflammatory Cytokines

Colonic IL-6, IL-1β and TNF-α concentrations were measured using double-antibody sandwich ELISA kits (IL-6: EK0411, IL-1β: EK0394, TNF-α: EK0527, BosterBio, Wuhan, Hubei, China) following the manufacturer’s instructions. Absorbance was read at 450 nm, and cytokine levels were calculated against standard curves run on each plate.

### 2.10. Quantitative Real-Time PCR (qPCR)

Total RNA was extracted from colonic mucosa using TRIzol reagent (15596026, Thermo Fisher Scientific, Waltham, MA, USA). RNA purity and concentration were assessed with a Nanodrop spectrophotometer (Thermo Fisher Scientific, Waltham, MA, USA). cDNA was synthesized by reverse transcription using PrimeScript RT Master Mix (RR037A, TaKaRa, Kusatsu, Shiga, Japan). qPCR was performed on a QuantStudio system (Thermo Fisher Scientific, Waltham, MA, USA) using TB Green Premix Ex Taq II (RR820A, TaKaRa, Kusatsu, Shiga, Japan) with the following cycling conditions: 95 °C for 30 s, followed by 40 cycles of 95 °C for 5 s and 60 °C for 10 s. Gapdh served as the internal reference. Relative gene expression was calculated using the 2^−ΔΔCt^ method. Primer sequences are listed in Table 2.

### 2.11. Western-Blot Analysis

Colonic mucosa samples were lysed in RIPA buffer (P0013B, Beyotime, Shanghai, China) supplemented with PMSF (ST506, Beyotime, Shanghai, China) and phosphatase inhibitors (P1082, Beyotime, Shanghai, China). Protein concentrations were determined using a BCA kit (P0010, Beyotime, Shanghai, China). Equal amounts of denatured protein (20 µg per lane) were resolved by 8% SDS-PAGE and transferred onto PVDF membranes (IPVH00010, Millipore, Burlington, MA, USA). Membranes were blocked with 5% non-fat milk in TBST for 2 h at room temperature, then incubated overnight at 4 °C with primary antibodies against PI3K (PA5-79765, 1:1000), p-PI3K (PA5-104722, 1:1000), Akt (PA5-79616, 1:1000), p-Akt (PA5-79617, 1:1000), FOXO3A (PA5-79912, 1:1000), p-FOXO3A (Ser253) (PA5-80325, 1:1000) and GAPDH (PA1-987, 1:5000; all from Thermo Fisher Scientific, Waltham, MA, USA). After washing, blots were incubated with HRP-conjugated secondary antibody (31460, Thermo Fisher Scientific, Waltham, MA, USA, 1:3000) for 1 h at room temperature. Protein bands were visualized using ECL reagent (34095, Thermo Fisher Scientific, Waltham, MA, USA) and imaged with an iBright CL1500 imaging system (Thermo Fisher Scientific, Waltham, MA, USA). Band intensities were quantified with ImageJ (version 1.53, NIH, Bethesda, MD, USA) and normalized to GAPDH.

### 2.12. Network-Pharmacology Analysis

Potential bioactive components of *Fagopyrum cymosum* were retrieved from the TCMSP database (https://www.tcmsp-e.com/ (accessed on 12 September 2025)) with screening criteria of oral bioavailability (OB) ≥ 30% and drug-likeness (DL) ≥ 0.18. Putative targets for these components were obtained from the SEA database (https://sea.bkslab.org/ (accessed on 12 September 2025)). UC-associated disease targets were collected from the GeneCards (https://www.genecards.org/ (accessed on 12 September 2025)) and OMIM (https://www.omim.org/ (accessed on 12 September 2025)) databases. Intersecting targets between the predicted drug targets and disease targets were imported into STRING 11.5 (https://string-db.org/ (accessed on 12 September 2025)) for PPI network construction with a confidence score threshold > 0.7. GO functional enrichment and KEGG pathway enrichment analyses were performed using the DAVID database (https://davidbioinformatics.nih.gov/ (accessed on 12 September 2025)), with *p* < 0.05 as the significance threshold. Bubble plots were generated using the R package ggplot2 (version 3.5.1; R Foundation for Statistical Computing, Vienna, Austria).

### 2.13. Statistical Analysis

Statistical analyses were carried out using SPSS 26.0 (IBM, Armonk, NY, USA), and figures were prepared with GraphPad Prism 9 (GraphPad Software, San Diego, CA, USA). All data are presented as mean ± SD. Body weight and DAI were analyzed using one-way ANOVA at each time point followed by Tukey’s post-hoc test. Other endpoint measures were analyzed using one-way ANOVA followed by Tukey’s HSD post-hoc test. For data that violated normality or homogeneity of variance assumptions, non-parametric tests were used. A *p*-value < 0.05 was considered statistically significant, and *p* < 0.01 was regarded as highly significant.

## 3. Results

### 3.1. RFC Protects Mice Against DSS-Evoked Ulcerative Colitis

**Figure 1:**
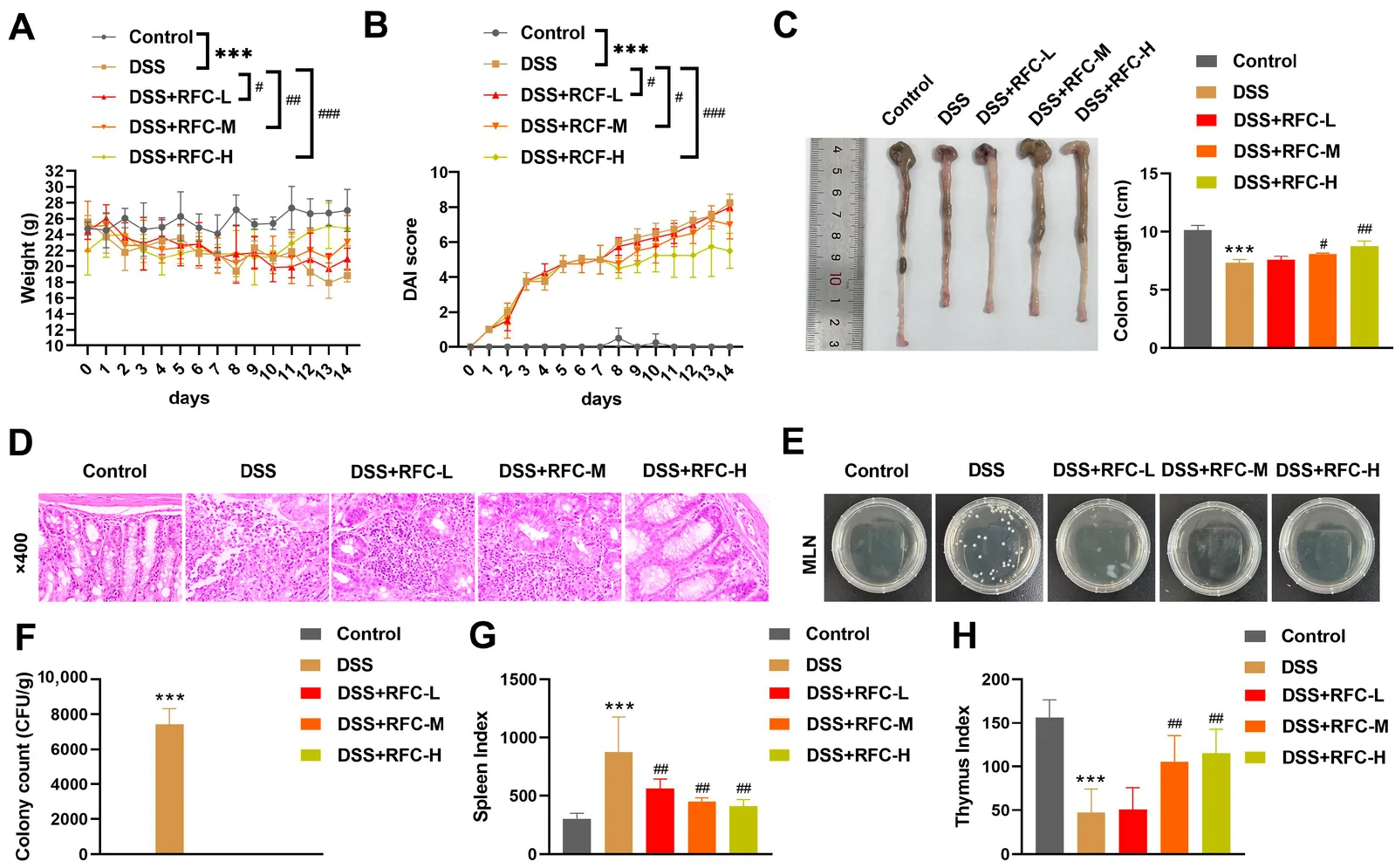
RFC exerts protective effects on DSS-induced ulcerative colitis in mice. (**A**) Body weight changes; (**B**) DAI curves; (**C**) Colon photographs and quantitative colon-length statistics; (**D**) HE staining of colon tissues (magnification ×400); (**E**) Bacterial colonies from MLNs; (**F**) Bacterial counts in MLNs; (**G**) spleen index; (**H**) thymus index. *** *p* < 0.001 vs. Control group; # *p* < 0.05, ## *p* < 0.01, and ### *p* < 0.001 vs. DSS group.

Compared with the control group, DSS-treated mice displayed marked body-weight loss and higher disease-activity index (DAI) scores. RFC treatment alleviated body-weight reduction and reduced DAI scores in a dose-dependent manner (Figure 1A,B). DSS exposure caused obvious colon shortening, and RFC administration restored colon length dose-dependently (Figure 1C). Hematoxylin-eosin (HE) staining of colon tissue showed extensive inflammatory cell infiltration, disrupted crypt architecture, and goblet-cell loss in the DSS group. RFC intervention attenuated mucosal damage, reduced inflammatory infiltration, and preserved crypt structure (Figure 1D). Bacterial-colony assays detected substantial bacterial translocation to mesenteric lymph nodes (MLNs) in DSS-exposed animals; RFC lowered bacterial translocation loads (Figure 1E,F). Moreover, DSS challenge increased the spleen index and decreased the thymus index, while RFC partly restored these immune-organ parameters (Figure 1G,H). Taken together, RFC produced dose-dependent protective effects against DSS-induced ulcerative colitis in mice.

### 3.2. RFC Ameliorates Colonic Mitochondrial Dysfunction, Oxidative Stress Imbalance and Pro-Inflammatory Cytokine Production

**Figure 2:**
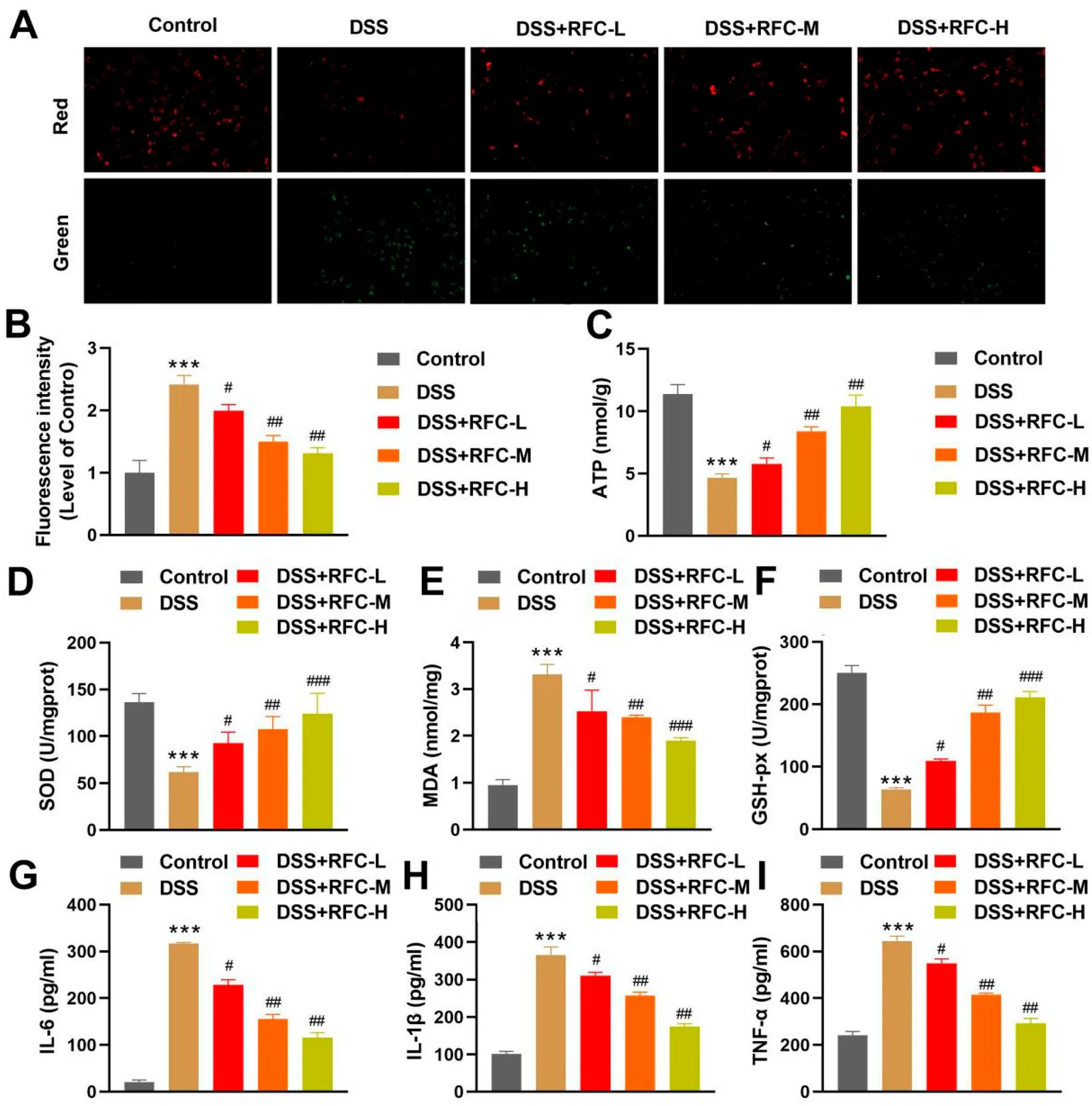
RFC improves mitochondrial function, relieves oxidative stress and suppresses pro-inflammatory cytokine secretion in colon tissue. (**A**) Representative JC-1 fluorescence images for mitochondrial-membrane-potential detection; (**B**) semi-quantification of the green/red fluorescence intensity ratio; (**C**) colonic ATP level; (**D**) SOD activity; (**E**) MDA content; (**F**) GSH-px activity; (**G**) IL-6; (**H**) IL-1β; (**I**) TNF-α concentrations determined by ELISA. Data are presented as mean ± SD (*n* = 6 per group). *** *p* < 0.001 vs. Control group; # *p* < 0.05, ## *p* < 0.01, and ### *p* < 0.001 vs. DSS group.

In DSS-treated mice, JC-1 staining revealed increased green fluorescence and decreased red fluorescence in colonic epithelial cells, indicating loss of mitochondrial membrane potential. RFC treatment reduced the green-to-red fluorescence ratio in a dose-dependent manner, suggesting partial restoration of MMP (Figure 2A,B). Colonic ATP content, which was markedly lower in DSS mice, was progressively recovered by RFC (Figure 2C). Regarding oxidative stress, DSS reduced SOD and GSH-px activities and elevated MDA levels; these alterations were counteracted by RFC in a dose-responsive fashion (Figure 2D–F). Similarly, DSS strongly induced colonic IL-6, IL-1β and TNF-α production, whereas RFC administration suppressed the release of these pro-inflammatory cytokines (Figure 2G–I).

### 3.3. Network-Pharmacology Prediction and Molecular Validation of RFC Targeting the PI3K/Akt/FOXO3A Signaling Axis

**Figure 3:**
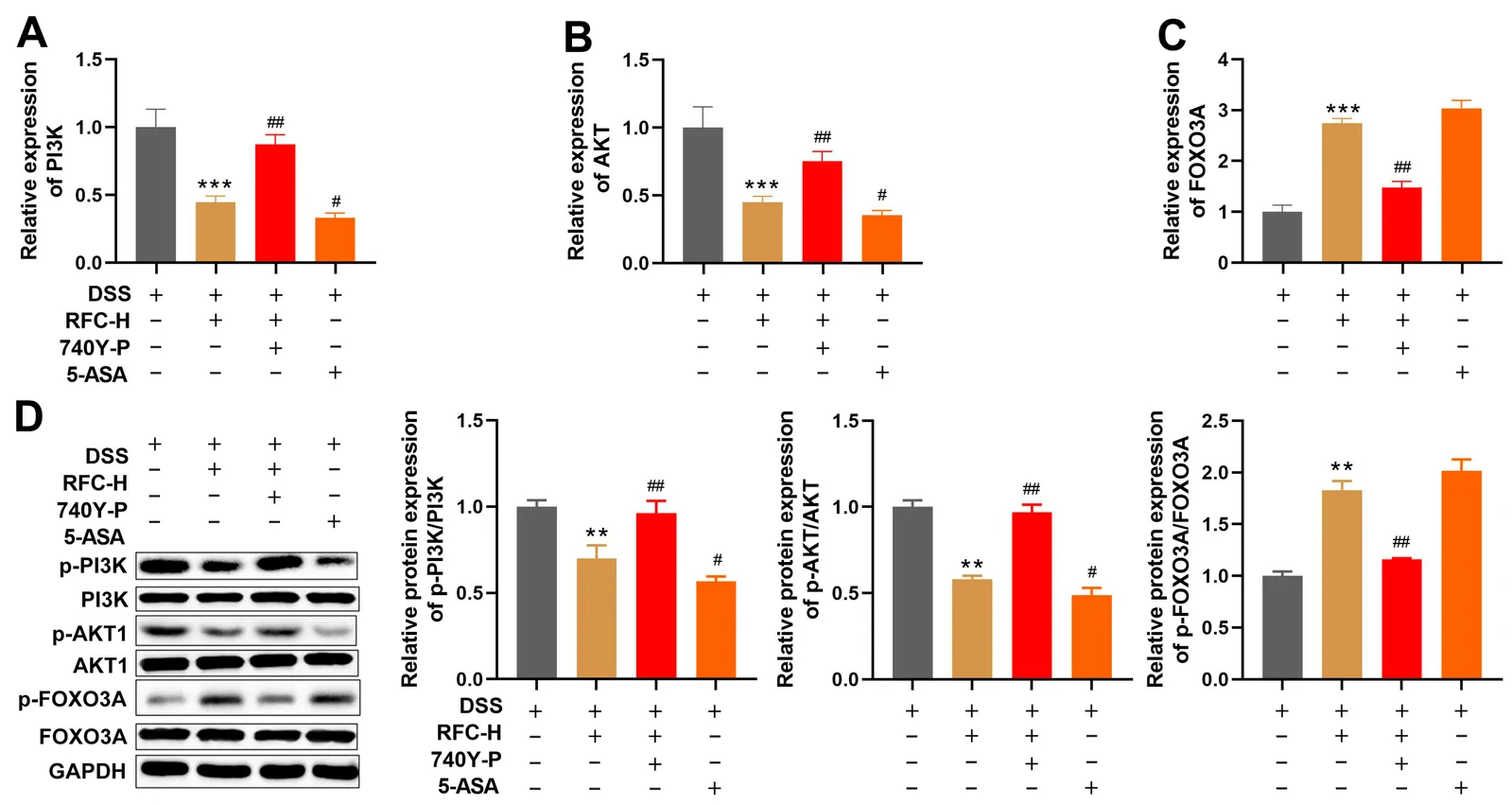
Molecular verification of RFC-mediated regulation of the PI3K/Akt/FOXO3A axis. (**A**–**C**) Relative mRNA expression of Pik3r1, Akt1 and Foxo3 determined by qPCR. (**D**) Representative Western blot bands and quantitative densitometry of total and phosphorylated PI3K, Akt and FOXO3A proteins. The high dose of RFC (RFC-H) was selected for mechanistic analysis based on the dose-dependent effects observed in Figure 1 and Figure 2. Data are presented as mean ± SD (*n* = 6 per group). ** *p* < 0.01 and *** *p* < 0.001 vs. DSS group; # *p* < 0.05 and ## *p* < 0.01 vs. DSS + RFC-H group. The high dose of RFC (RFC-H) was selected for mechanistic analysis based on the dose-dependent protective effects observed in Figure 1 and Figure 2. The Control group data are presented in Figure 1 and Figure 2 and are not repeated here.

Five candidate bioactive components of *Fagopyrum cymosum* (beta-sitosterol, sitosterol, digallate, coumaroyltyramine and (−)-catechin gallate) were retrieved from the TCMSP database for target prediction. Among these, only (−)-catechin gallate and gallate-containing proanthocyanidins were experimentally detected in the RFC batch by MS profiling (Section 2.2), whereas the sterol-type candidates were not identified under the current conditions. A protein–protein interaction (PPI) network was constructed for the overlapping targets between RFC-predicted components and UC-associated disease genes, and GO and KEGG enrichment analyses showed significant target enrichment in PI3K/Akt, oxidative stress and inflammatory regulatory pathways (Supplementary Figure S2A,B). In line with this prediction, qPCR revealed that high-dose RFC down-regulated Pik3r1 and Akt1 mRNA and up-regulated Foxo3 mRNA in colonic tissues; these transcriptional changes were largely reversed by co-administration of the PI3K agonist 740Y-P (Figure 3A–C). Western blotting further confirmed that RFC-H decreased the phosphorylation levels of PI3K and Akt, while it increased FOXO3A phosphorylation. The addition of 740Y-P antagonized all these phosphorylation changes. RFC-H exhibited regulatory effects comparable to those of the positive control 5-ASA (Figure 3D).

### 3.4. PI3K Agonist 740Y-P Partially Reverses RFC-Mediated Protection Against UC

**Figure 4:**
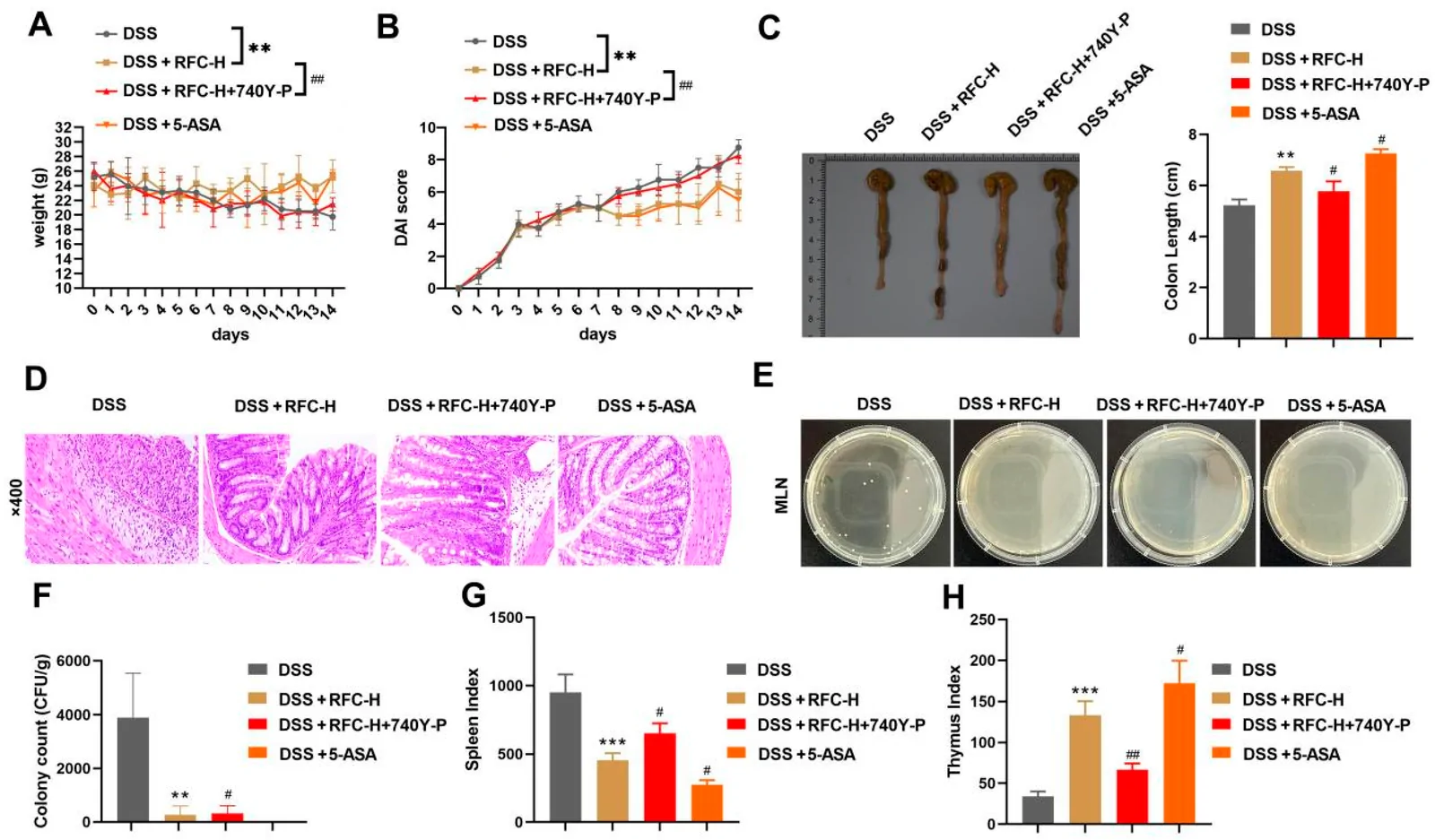
The PI3K-specific agonist 740Y-P reverses RFC-afforded protection in DSS-induced colitis mice. (**A**) Body-weight changes. (**B**) DAI score curves. (**C**) Colon length statistics and representative colon images. (**D**) HE-stained colon sections (scale bar: 50 μm). (**E**) Representative MLN bacterial-culture plates; the Control group image is shown in Figure 1E. (**F**) MLN bacterial counts. (**G**) Spleen index. (**H**) Thymus index. Data are presented as mean ± SD (*n* = 6 per group). ** *p* < 0.01 and *** *p* < 0.001 vs. DSS group; # *p* < 0.05 and ## *p* < 0.01 vs. DSS + RFC-H group. The Control group data are presented in Figure 1 and Figure 2 and are not repeated here.

High-dose RFC administration improved body-weight recovery and reduced DAI scores in DSS-treated mice; these effects were partially reversed by co-treatment with the PI3K agonist 740Y-P (Figure 4A,B). Colon shortening was similarly attenuated by RFC, an effect counteracted by 740Y-P (Figure 4C). Histologically, RFC repaired colonic mucosal injury and diminished inflammatory infiltration, whereas addition of 740Y-P markedly impaired this mucosal repair (Figure 4D). RFC also reduced bacterial translocation to mesenteric lymph nodes, an effect that was abolished by PI3K activation (Figure 4E,F). In addition, 740Y-P reversed the RFC-induced recovery of spleen and thymus indices (Figure 4G,H). Across all parameters, high-dose RFC performed comparably to the positive control 5-ASA.

### 3.5. PI3K Agonist 740Y-P Reverses RFC-Mediated Mitochondrial Protection as Well as Antioxidant and Anti-Inflammatory Effects

**Figure 5:**
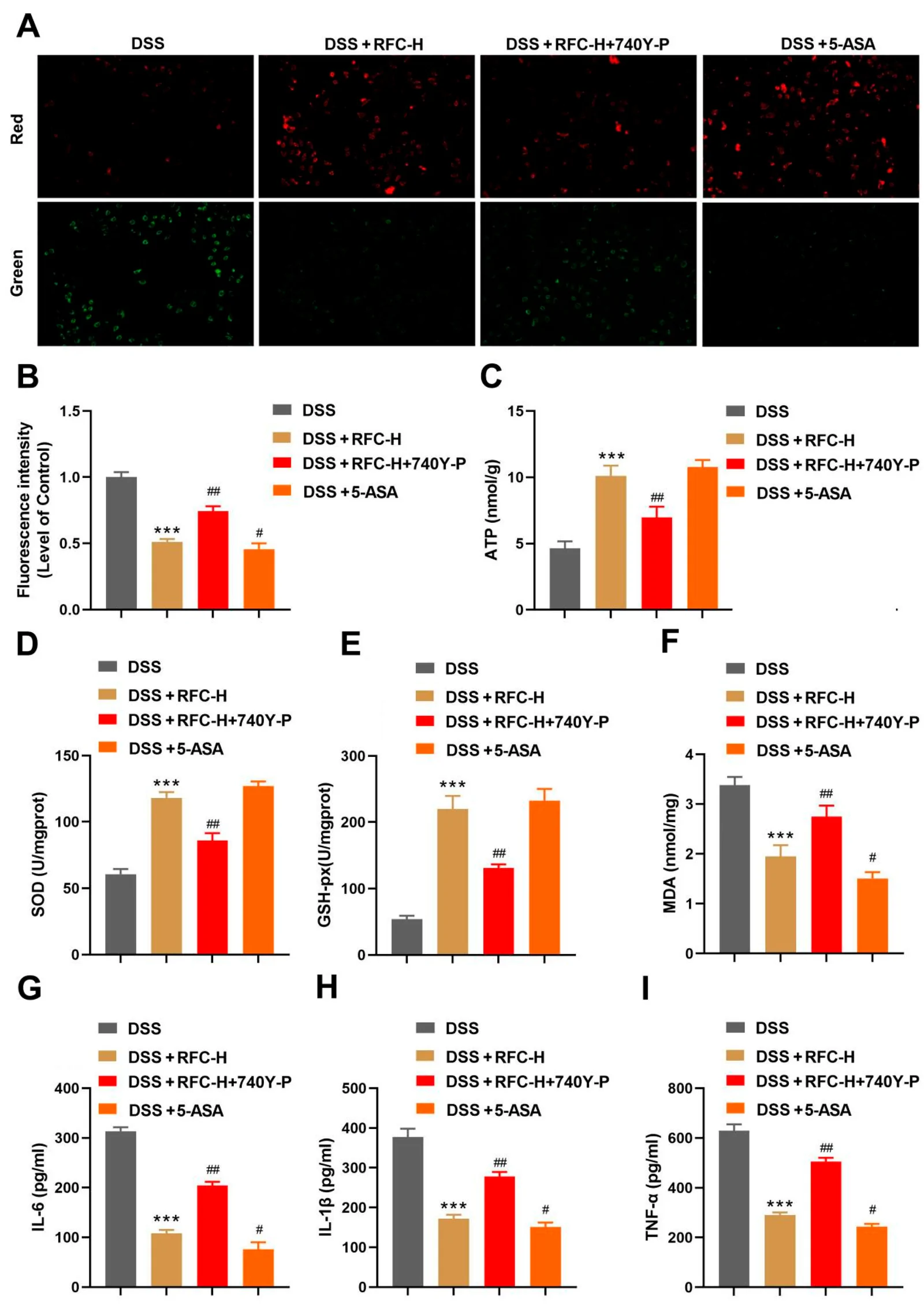
The PI3K agonist 740Y-P reverses RFC-mediated mitochondrial protection as well as antioxidant and anti-inflammatory effects. (**A**) Representative JC-1 fluorescence images for mitochondrial-membrane-potential detection. (**B**) Quantitative green/red fluorescence intensity ratio. (**C**) Colonic ATP level. (**D**) SOD activity. (**E**) GSH-px activity. (**F**) MDA content. (**G**–**I**) Colonic IL-6, IL-1β and TNF-α concentrations determined by ELISA. Data are presented as mean ± SD (*n* = 6 per group). *** *p* < 0.001 vs. DSS group; # *p* < 0.05 and ## *p* < 0.01 vs. DSS + RFC-H group. The Control group data are presented in Figure 1 and Figure 2 and are not repeated here.

JC-1 staining showed that high-dose RFC preserved the mitochondrial membrane potential in colonic epithelial cells, whereas co-treatment with 740Y-P attenuated this protective effect (Figure 5A,B). The RFC-induced increase in colonic ATP content was similarly blunted by the PI3K agonist (Figure 5C). RFC-H elevated SOD and GSH-px activities and reduced MDA accumulation; these redox modifications were largely suppressed in the presence of 740Y-P (Figure 5D–F). Likewise, the RFC-mediated suppression of IL-6, IL-1β and TNF-α production was counteracted by PI3K activation (Figure 5G–I). Across all parameters, high-dose RFC performed comparably to the positive control 5-ASA.

## 4. Discussion

Ulcerative colitis is a chronic inflammatory bowel disease driven by immune dysregulation and metabolic disturbances [22]. *Fagopyrum cymosum* has long been used for acute lung abscess and bronchitis owing to its anti-inflammatory, antioxidant and antimicrobial properties [23]. A previous study using TNBS-induced colitis showed that *F. cymosum* extract ameliorated body weight loss and colonic shortening, suppressed TNF-α and IL-6 production, and inhibited NF-κB p65 nuclear translocation and IκB phosphorylation [6]. In vitro, the same extract suppressed TNF-α and IL-6 production in LPS-stimulated RAW264.7 macrophages through inhibition of NF-κB signaling [6]. These findings support the therapeutic potential of *F. cymosum* in UC. However, direct evidence for its efficacy in DSS-induced colitis and the underlying mechanism remained lacking. Here, we show that RFC dose-dependently ameliorated the cardinal features of DSS-induced colitis—weight loss, colonic injury, bacterial translocation and immune organ dysregulation—establishing its protective effect in this model.

The doses of RFC used here (234, 468, and 936 mg/kg) were selected based on previously reported effective doses of *F. cymosum* extracts in rodent colitis models [6] and available acute toxicity data. The LD50 of *F. cymosum* extract exceeds 5.0 g/kg in mice, which is classified as non-toxic. A maximum tolerated dose of 8.0 g/kg without mortality has also been reported. In subchronic toxicity studies, high doses (34–40 g/kg) affected liver and kidney structure in rats, but these doses are 36–43 times higher than the highest dose used in the present study. These data support the safety of RFC at the doses tested, although long-term toxicity studies are warranted before clinical translation.

Mitochondria lie at the center of cellular redox control. Excessive oxidative stress and reactive oxygen species damage mitochondrial function, which in turn fuels intestinal inflammation [24,25]. Our data show that RFC rescues DSS-induced mitochondrial dysfunction, as indicated by preserved mitochondrial membrane potential and ATP content, reduced MDA accumulation, and elevated SOD and GSH-px activities. The chemical profiling of RFC revealed a complex mixture of polyphenols and flavonoids, including epicatechin, procyanidin B2, kaempferol glycosides, gallic acid derivatives, and syringic acid. Epicatechin and procyanidin B2 have been reported to directly modulate mitochondrial respiration and membrane stability, and to improve mitochondrial dynamics via Nrf2. These findings suggest that the mitochondrial protection afforded by RFC may be attributed, at least in part, to its polyphenolic constituents. Epicatechin and procyanidin B2 have also been reported to preserve mitochondrial membrane fluidity, reduce reactive oxygen species accumulation, and regulate mitochondrial fission and fusion. Whether these mechanisms contribute to the effects of RFC in DSS-induced colitis requires further investigation. Although free quercetin aglycone was not detected, its glycosylated derivatives such as rutin were present, and these can be deglycosylated by intestinal enzymes to release active aglycones after oral administration.

Network pharmacology identified the PI3K/Akt axis as a top candidate pathway. This cascade modulates intestinal inflammation, redox status and epithelial barrier integrity in UC [9,10]. FOXO3A, a downstream transcription factor negatively regulated by PI3K/Akt-mediated phosphorylation [11], is downregulated in colonic tissue from UC patients and inactivated in DSS-treated mice [11,26,27]. In our study, RFC suppressed PI3K/Akt phosphorylation and upregulated Foxo3 mRNA and FOXO3A total protein. We note that RFC-H increased FOXO3A phosphorylation at Ser253, a site phosphorylated by Akt that promotes FOXO3A nuclear export. This may appear inconsistent with the sustained antioxidant enzyme activities observed. However, the concurrent upregulation of Foxo3 mRNA and FOXO3A total protein suggests that RFC may maintain FOXO3A transcriptional capacity through increased expression rather than through dephosphorylation. One possible explanation is that RFC, as a complex extract, regulates FOXO3A through multiple mechanisms, and that Ser253 phosphorylation alone may not fully reflect FOXO3A transcriptional activity. The net effect of RFC on PI3K/Akt signaling may also reflect the combined actions of multiple constituents. Quercetin, a flavonoid present in *F. cymosum*, inhibits PI3K/Akt phosphorylation, whereas epicatechin has been reported to activate PI3K/Akt in some contexts. The observed reduction in PI3K/Akt phosphorylation in our study suggests that the inhibitory constituents may predominate in this model. In rescue experiments, the PI3K agonist 740Y-P attenuated these effects and largely reversed the beneficial actions of RFC, confirming that the protection is at least in part PI3K/Akt/FOXO3A-dependent.

Bacterial translocation to mesenteric lymph nodes is a hallmark of impaired intestinal barrier function in UC. In our study, DSS exposure led to substantial bacterial translocation, and RFC reduced the bacterial load in MLNs. This is consistent with a previous report that *F. cymosum* capsules restore tight junction protein expression in DSS-induced colitis. Although we did not directly measure tight junction protein expression, the reduction in bacterial translocation suggests that RFC may protect epithelial barrier integrity. Future studies should examine the effects of RFC on tight junction proteins and intestinal permeability.

Current targeted therapies for UC include JAK inhibitors such as tofacitinib, filgotinib, and upadacitinib. Real-world data show that these agents achieve clinical remission rates of approximately 40–60% at 24 weeks, with filgotinib demonstrating favorable remission rates in biologic-naïve patients and upadacitinib showing the highest clinical response at week 8. However, JAK inhibitor recipients experience an approximately 4-fold higher incidence of adverse events compared with vedolizumab and ustekinumab, and safety concerns warrant careful patient selection. The mechanism of RFC differs from that of JAK inhibitors: RFC targets the PI3K/Akt/FOXO3A axis, whereas JAK inhibitors block JAK-STAT signaling. These pathways are distinct but functionally interconnected. Combined inhibition of PI3K/AKT and JAK2/STAT3 has been shown to exert synergistic anti-colitis effects in preclinical models, suggesting that RFC could potentially complement JAK inhibitor therapy. Given the safety concerns associated with long-term JAK inhibitor use, natural products such as RFC may offer a favorable safety profile for maintenance therapy or combination regimens, although this requires formal toxicity evaluation.

A further consideration for RFC development is its delivery form. As a crude extract, RFC may undergo gastrointestinal degradation, limited absorption, and insufficient accumulation at inflamed sites. Nano-formulation strategies could address these issues. Chemotaxis-driven hybrid liposomes have been used for targeted colonic delivery in colitis models [28], and thiol-functionalized nanoliposomes loaded with gallic acid, a constituent detected in our RFC batch, showed prolonged retention and superior efficacy compared with free gallic acid [29]. Whether nano-formulated RFC improves colonic targeting and release kinetics requires further study.

Our findings are based on a murine DSS colitis model, which may not fully reflect human UC. Patient-derived intestinal organoids retain donor-specific epithelial characteristics and have been used to evaluate drug responses in IBD. A recent study found that organoid responses to SIK2/3 inhibition aligned with phase 2 clinical trial data [30], and another showed that patient-derived organoids predict clinical responsiveness to tofacitinib in UC patients [31]. Future studies should test whether RFC modulates barrier integrity, mitochondrial function, and PI3K/Akt/FOXO3A signaling in UC patient-derived organoids.

Several limitations should be acknowledged. First, the functional data are derived solely from mouse experiments, which may not fully recapitulate human UC pathophysiology; validation in human cells or organoids is needed before clinical translation. Second, the chemical constituents of RFC were tentatively annotated but not quantified with reference standards, so the relative contribution of each compound to the observed effects cannot be determined. Third, the network pharmacology targets are computational predictions requiring experimental validation; the PI3K/Akt/FOXO3A axis should be confirmed by additional approaches such as pathway inhibitors or gene silencing. Fourth, long-term safety data are lacking, and chronic toxicity studies are needed to evaluate the safety of RFC for prolonged use. Fifth, the delivery form of RFC was not optimized; nano-formulation strategies may improve colonic targeting and release kinetics. Sixth, JC-1 imaging was performed with an inverted fluorescence microscope rather than confocal microscopy, which limits the spatial resolution of mitochondrial membrane potential analysis. Future work should isolate the principal bioactive constituents, validate efficacy in patient-derived organoids, explore nano-formulation strategies, and conduct chronic toxicity studies.

## 5. Conclusions

In summary, this study demonstrates that *Fagopyrum cymosum* extract (RFC) exerts dose-dependent protective effects against DSS-induced ulcerative colitis in mice. RFC improved body weight loss, reduced DAI scores, restored colon length, attenuated mucosal damage, decreased bacterial translocation to mesenteric lymph nodes, and normalized spleen and thymus indices. At the mitochondrial level, RFC preserved mitochondrial membrane potential and ATP content, reduced MDA accumulation, and elevated SOD and GSH-px activities. RFC also suppressed colonic IL-6, IL-1β, and TNF-α production. Mechanistically, network pharmacology predicted the PI3K/Akt/FOXO3A axis as a top enriched pathway, and experimental validation showed that RFC inhibited PI3K/Akt phosphorylation and upregulated FOXO3A expression. The PI3K agonist 740Y-P reversed these effects and abolished the protective, antioxidant, and anti-inflammatory actions of RFC, confirming that the protection is at least in part PI3K/Akt/FOXO3A-dependent. The PI3K/Akt/FOXO3A axis therefore represents a functionally relevant node for therapeutic intervention in UC, and RFC merits further evaluation as a candidate for clinical development. Future studies should focus on isolating and characterizing the principal bioactive constituents of RFC, validating its efficacy in patient-derived intestinal organoids, exploring nano-formulation strategies to improve colonic targeting and release kinetics, and evaluating its long-term safety in chronic toxicity studies.

## Figures and Tables

**Table 1:** DAI scoring criteria for mice.

Score	Body-Weight Loss (%)	Fecal Consistency	Fecal Occult Blood
0	<1	Normal formed stool	Negative (no bleeding)
1	1–5	Soft stool	Occult blood positive (+)
2	5–10	Wet stool	Slight hematochezia (2+)
3	10–15	Loose stool	Partial hematochezia (3+)
4	>15	Watery stool	Massive hematochezia (4+)

Fecal occult blood was determined by the o-tolidine colorimetric assay.

**Table 2:** Primer sequences used for qPCR.

Gene	Forward Primer (5′ → 3′)	Reverse Primer (5′ → 3′)
*Pik3r1*	CCCTCAGTGGACTTGGATGT	GCTGCTGGGAATCTGAAAAG
*Akt1*	ACTCATTCCAGACCCACGAC	TGAGCTCGAACAGCTTCTCA
*Foxo3*	TGCTAAGCAGGCCTCATCTTC	GGTGCGTTGCGTGCCCTACT
*Gapdh*	CCGTCTAGAAAAACCTGCC	AGCCAAATTCGTTGTCATACC

## Data Availability

The raw data generated in this study are available from the corresponding authors upon request.

## Supplementary Materials

The following supporting information can be downloaded at: https://www.mdpi.com/article/10.3390/biomedicines14102403/s1; Figure S1: Total ion current chromatograms of the RFC extract obtained by UHPLC-Q-TOF-MS/MS in (A) positive-ion and (B) negative-ion modes. Figure S2: Network pharmacology analysis of RFC targets. (A) PPI network of overlapping targets between RFC-predicted components and UC-associated genes. (B) GO and KEGG enrichment bubble plots. Table S1: List of tentatively annotated chemical constituents in the RFC extract. A. Positive-ion mode. B. Negative-ion mode.

## Abbreviations

The following abbreviations are used in this manuscript:

Abbreviation	Full Name
UC	Ulcerative colitis
RFC	*Fagopyrum cymosum* extract
DSS	Dextran sulfate sodium
DAI	Disease-activity index
MMP	Mitochondrial membrane potential
MLN	Mesenteric lymph nodes
SOD	Superoxide dismutase
GSH-px	Glutathione peroxidase
MDA	Malondialdehyde
ELISA	Enzyme-linked immunosorbent assay
qPCR	Quantitative real-time polymerase chain reaction
PPI	Protein–protein interaction
GO	Gene Ontology
KEGG	Kyoto Encyclopedia of Genes and Genomes
5-ASA	5-Aminosalicylic acid
TCMSP	Traditional Chinese Medicine Systems Pharmacology Database
