# Supplementary material for: Fagopyrum cymosum Extract Improves Mitochondrial Function and Reduces Colonic Inflammation in Ulcerative Colitis by Targeting the PI3K/Akt/FOXO3A Pathway
Source: Biomedicines. 2026 Oct 21;14(10):2403. doi: 10.3390/biomedicines14102403 (PMC13701870; doi:10.3390/biomedicines14102403)
Supplement: Supplementary file 1 [file biomedicines-14-02403-s001.zip › Figures S1 and S2.pdf]

Supplementary materials

Fagopyrum cymosum Extract Improves Mitochondrial Function and Reduces Colonic Inflammation in Ulcerative Colitis by Targeting the PI3K/Akt/FOXO3A Pathway

Shanshan Xu [1], Congtian Fan [1], Haidong Chen [1], Dan Chen [1], Jie Lu [1], Jianyong Xu [1], Hua Huang [2,*] and Yu Jiang [1,*]

1 *Nantong Hospital Affiliated to Nanjing University of Chinese Medicine, Nantong, 226001, China; symphoni33@163.com (S.X.)*

2 *Changshu Hospital Affiliated to Nanjing University of Chinese Medicine, Changshu, 215500, China*

* **Correspondence:** fsyy01041@njucm.edu.cn (H.H.); 15962982720@163.com (Y.J.)

Supplementary Figures and legends

A

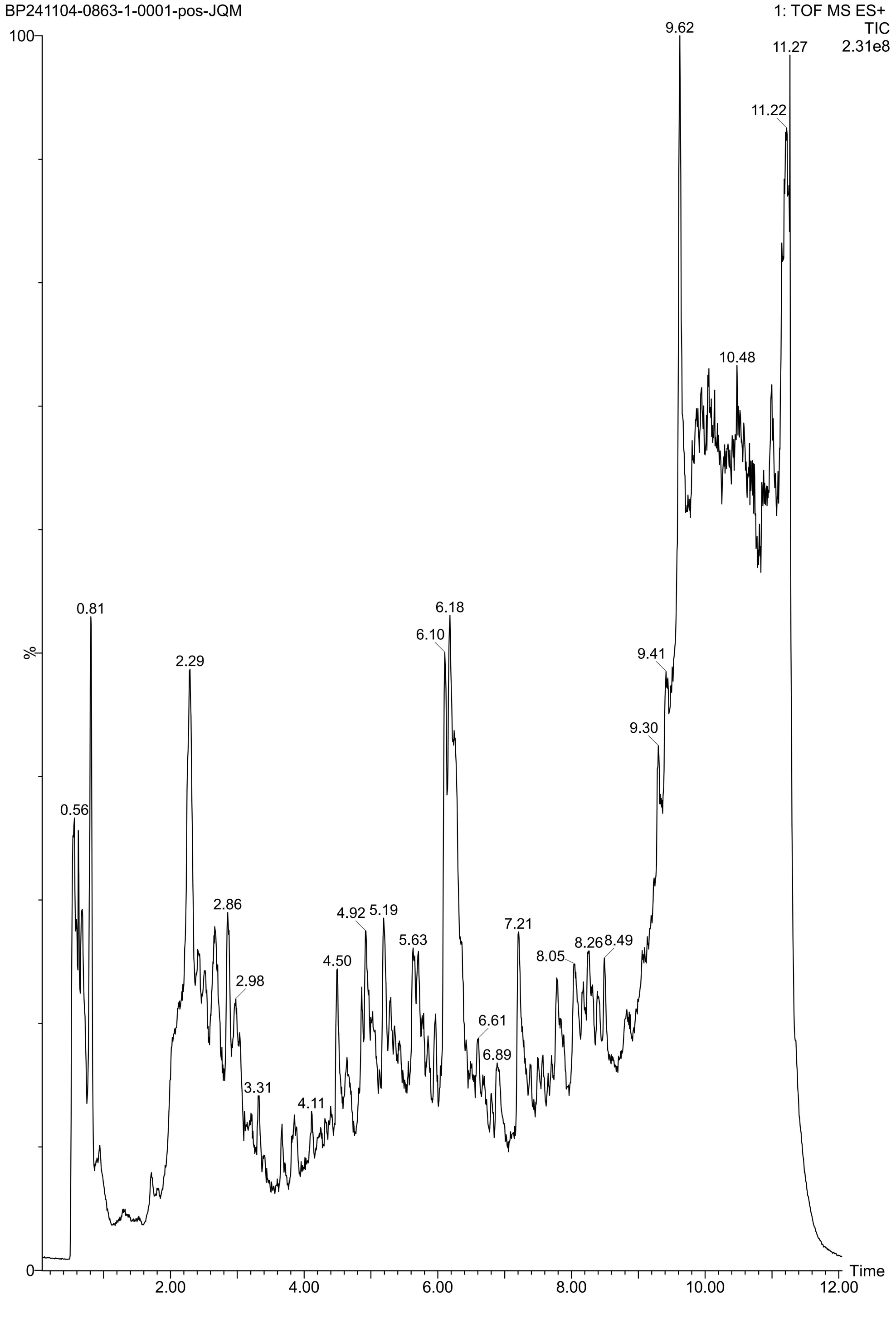

B

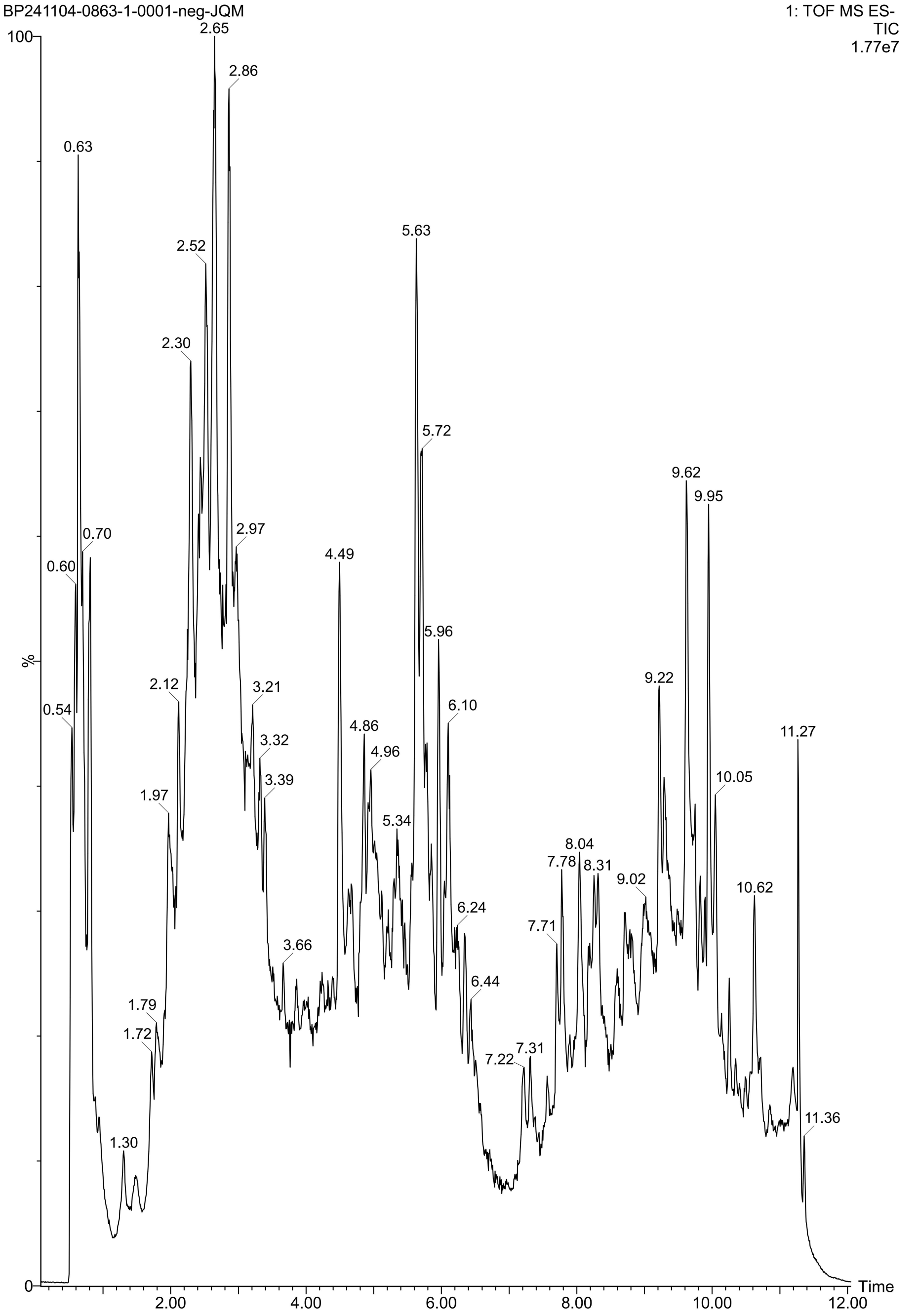

Figure S1. Total ion current chromatograms of the RFC extract obtained by UHPLC-Q-TOF-MS/MS in (A) positive-ion and (B) negative-ion modes.

A

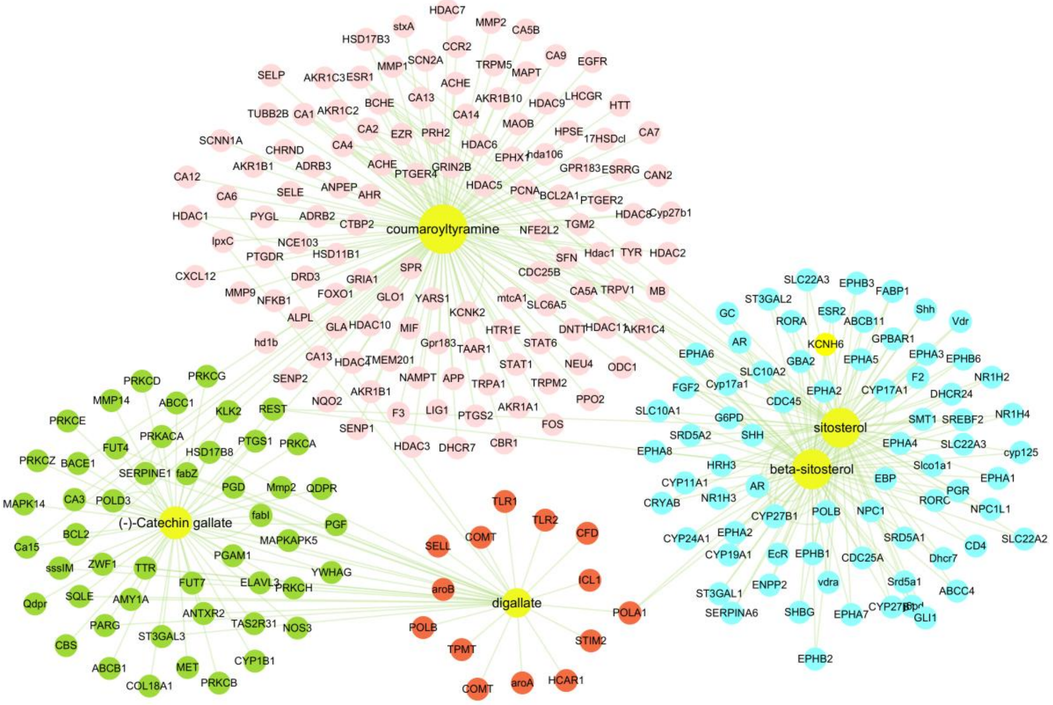

B

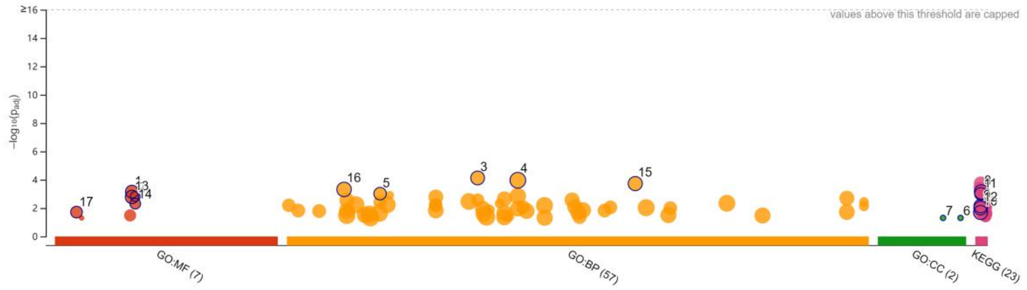

ID	Source	Term ID	Term Name	p_{adj} (query_1)
1	GO:MF	GO:0019903	protein phosphatase binding	6.936×10^{-4}
2	GO:MF	GO:0030297	transmembrane receptor protein tyrosine kinase activator activity	1.510×10^{-3}
3	GO:BP	GO:0034599	cellular response to oxidative stress	7.449×10^{-5}
4	GO:BP	GO:0043069	negative regulation of programmed cell death	1.084×10^{-4}
5	GO:BP	GO:0010595	positive regulation of endothelial cell migration	9.522×10^{-4}
6	GO:CC	GO:1990150	VEGF-A complex	4.915×10^{-2}
7	GO:CC	GO:0097489	multivesicular body, internal vesicle lumen	4.915×10^{-2}
8	KEGG	KEGG:04151	PI3K-Akt signaling pathway	7.228×10^{-4}
9	KEGG	KEGG:04015	Rap1 signaling pathway	7.065×10^{-3}
10	KEGG	KEGG:04010	MAPK signaling pathway	2.055×10^{-2}
11	KEGG	KEGG:04066	HIF-1 signaling pathway	9.926×10^{-4}
12	KEGG	KEGG:04014	Ras signaling pathway	9.871×10^{-3}
13	GO:MF	GO:0019902	phosphatase binding	1.630×10^{-3}
14	GO:MF	GO:0030296	protein tyrosine kinase activator activity	4.889×10^{-3}
15	GO:BP	GO:0062197	cellular response to chemical stress	1.858×10^{-4}
16	GO:BP	GO:0006979	response to oxidative stress	4.800×10^{-4}
17	GO:MF	GO:0004714	transmembrane receptor protein tyrosine kinase activity	1.919×10^{-2}

Figure S2. Network pharmacology analysis of RFC targets. (A) PPI network of overlapping targets between RFC-predicted components and UC-associated genes. (B) GO and KEGG enrichment bubble plots.
